# Supplementary material for: Contact-electro-catalytic CO2 reduction from ambient air
Source: Nat Commun. 2024 Jul 13;15:5913. doi: 10.1038/s41467-024-50118-1 (PMC11246423; doi:10.1038/s41467-024-50118-1)
Supplement: Supplementary file 1 — Supporting Information [file 41467_2024_50118_MOESM1_ESM.pdf]

Supplementary Information

Contact-electro-catalytic CO_2 reduction from ambient air

Nannan Wang[1‡], Wenbin Jiang[2‡], Jing Yang[3], Haisong Feng[3], Youbin Zheng[4], Sheng Wang[1], Bofan Li[1], Jerry Zhi Xiong Heng[1], Wai Chung Ong[1], Hui Ru TAN[2], Yong-Wei Zhang[3], Daoai Wang[5,6,*], Enyi Ye[2*], Zibiao Li[1,2,7,*]

[1] Institute of Sustainability for Chemicals, Energy and Environment (ISCE[2]), Agency for Science, Technology and Research (A*STAR), 1 Pesek Road, Jurong Island, Singapore 627833, Republic of Singapore;

[2] Institute of Materials Research and Engineering (IMRE), Agency for Science, Technology and Research (A*STAR), 2 Fusionopolis Way, Innovis #08-03, Singapore 138634, Republic of Singapore;

[3] Institute of High Performance Computing (IHPC), Agency for Science, Technology and Research (A*STAR), 1 Fusionopolis Way, #16-16 Connexis, Singapore 138632, Republic of Singapore;

[4] Department of Electrical Engineering & Electronics, University of Liverpool, Brownlow Hill, Liverpool, L69 7GJ, UK;

[5] State Key Laboratory of Solid Lubrication, Lanzhou Institute of Chemical Physics, Chinese Academy of Sciences, Lanzhou 730000, China;

[6] Shandong Laboratory of Yantai Advanced Materials and Green Manufacturing, Yantai 265503, China;

[7] Department of Materials Science and Engineering, National University of Singapore, 9 Engineering Drive 1, Singapore 117576, Singapore.

‡ These authors contributed equally.

[*]Correspondence and requests for materials should be addressed to Z. L. (E-mail: lizb@imre.a-star.edu.sg), E.Y. (E-mail: yeey@imre.a-star.efu.sg) and D. W. (E-mail: wangda@licp.cas.cn)

Table of Contents

Supplementary Notes..4

Supplementary Note 1. Synthesis of quaternized CNF film.................................4

Supplementary Note 2. Simulated charge distribution near the PVDF and Cu-PCN surfaces during contact electrification. 5

Supplementary Note 3. The TENG working cycle, highlighting the integrated TENG charge generation and catalytic charge consumption process during contact-electro-catalytic CO_2RR..6

Supplementary Note 4. Synthesis of Cu-anchored polymeric carbon nitride (Cu-PCN). 7

Supplementary Note 5. EXAFS fitting parameters. 8

Supplementary Note 6. Moisture resistance performance and mechanism..........9

Supplementary Note 7. Comparison of output of pure CNF-Cu-PCN@PVDF based and quaternized CNF-Cu-PCN@PVDF based TENGs.............................10

Supplementary Note 8. Effect of high voltage generated by TENG on CO_2 dissociation. 11

Supplementary Note 9. Effect of tribolayer thickness on electrical output and CO_2RR of TENG. 12

Supplementary Note 10. Effect of tribolayer area on electrical output and CO_2RR of TENG...14

Supplementary Note 11. Device stability analysis. 15

Supplementary Note 12. Effect of high-humidity on CO_2 concentration...........16

Supplementary Note 13. Wind-driven TENG catalyzes CO_2 reduction in air....17

Supplementary Figures 19

Supplementary Tables 29

Supplementary Table 1 EXAFS fitting parameters at the Cu K-edge (S_0^2=0.88) 29

Supplementary Table 2 Analyzing CO yield and TENG current across 7 cycles using analysis of variance. 29

Supplementary Table 3 Quaternized CNF-Cu-PCN@PVDF based TENG

catalyzes CO_2RR in CO_2/Ar mixed gas with 20% CO_2 content. 29
Supplementary Table 4 Comparison of FE_{CO} and FE_{CO} between contact-electro-catalytic CO_2RR and state-of-the-art electrocatalytic CO_2RR using analysis of variance. 30
Supplementary Table 5 Quaternized CNF-Cu-PCN@PVDF based TENG catalyzes CO_2RR in air. 30
Supplementary References 31

Supplementary Notes

Supplementary Note 1. Synthesis of quaternized CNF film.

The procedure for synthesizing quaternized CNF was conducted based on the previous study[1], with slight modifications introduced. 30 g of aqueous CNF suspension was centrifuged and the water supernatant was removed. Then, 30 ml of acetone was added and the mixture was vortexed and centrifuged again to remove the acetone and residual water. The resulting solid CNF pellet was then dispersed in 100 ml DMSO and added to a round bottom flask. 1 g TBAH was added to the vessel and the contents were degassed under N_2 for 30 min. Then, 2.49 ml GTMAC was injected into the vessel and the temperature was increased to 65 °C. The product redispersed in water was centrifuged and the DMSO supernatant was removed. Then, 30 ml of acetone was added and the mixture was vortexed and centrifuged again to remove acetone and residual DMSO. The purified quaternized CNF pellet was then redispersed in water. The quaternized CNF film was obtained by suction filtration on a PVDF filter film with a pore size of 0.5 μm. The preparation process of quaternized CNF is shown in Supplementary Fig. 1a, and the photo of the quaternized CNF film is shown in Supplementary Fig. 1b. The SEM and TEM images of quaternized CNF is shown in Supplementary Fig. 1c.

Supplementary Note 2. Simulated charge distribution near the PVDF and Cu-PCN surfaces during contact electrification.

To intuitively demonstrate the transfer of electrons from PVDF to Cu-PCN, enabling Cu-PCN to establish an electric potential, DFT was employed to calculate the charge density difference of Cu-PCN@PVDF, as depicted in Supplementary Fig. 2 (Isosurfaces is 0.008 e $Å^{-3}$). In Supplementary Fig. 2a-c, yellow indicates the accumulation of electrons, while blue represents the consumption of charges. All calculations have been implemented in the VASP code by using the projector augmented wave method with the Perdew–Burke–Ernzerhof (PBE) exchange-correlation functional. Upon contact between PVDF and Cu-PCN, a clear electron transfer occurs from PVDF to Cu-PCN, as evidenced by the increased blue coloration on PVDF and increased yellow coloration on Cu-PCN. Furthermore, the yellow area on Cu atoms is more pronounced than that on other atoms, indicating that electrons from PVDF are primarily transferred to Cu atoms.

Supplementary Note 3. The TENG working cycle, highlighting the integrated TENG charge generation and catalytic charge consumption process during contact-electro-catalytic CO_2RR.

As depicted in Fig.1bi, the TENG device comprises two components, an electronegative tribolayer on the Aluminium (Al) back electrode (Cu-PCN@PVDF/Al) and an electropositive tribolayer on the Al back electrode (CNF/Al). The two Al back electrodes are connected via an external circuit with an ammeter attached to measure the electrical output of the TENG. The initial stage of contact electrification involves the complete contact of the two tribolayers, leading to the generation of triboelectric charges. When the two tribolayers are separated, induced electrons migrate from the Al back electrode of Cu-PCN@PVDF/Al to that of CNF/Al through the external circuit, indicating a current direction pointing from CNF/Al to Cu-PCN@PVDF/Al. Furthermore, CO_2 molecules can be captured by the quaternized CNF and interact with electrons stored in the Cu-PCN catalyst to initiate CO_2 reduction (Fig.1bii). In the meantime, water molecules in the high-humidity environment undergo oxidation on the surface of positively charged quaternized CNF. When the separation distance between the two tribolayers reaches the maximum, the two electrodes reach an electrical equilibrium state and no current flow (i.e., TENG's electrical output) can be detected in the external circuit (Fig.1biii). At this point, the induced charge on the two Al back electrodes also reaches its maximum level. Afterward, the Cu-PCN@PVDF/Al electrode is compressed downward, causing the induced electrons on the Al back electrode of CNF/Al to flow back toward that of the Cu-PCN@PVDF/Al (Fig.1biv). As the compression process advances, CO_2 molecules adsorbed on the quaternized CNF surface come into contact with Cu-PCN, facilitating the electron transfer for CO_2 reduction (Fig.1bv). Concurrently, water molecules undergo oxidation to complete the catalytic cycle. Subsequently, the charge in the tribolayers of the TENG is exhausted, and the two electrodes reach another equilibrium state with no current flow in the external circuit (Fig.1bvi). The electrical output of the TENG initiates the next identical working cycle once the two tribolayers make complete contact and triboelectric charges are generated.

Supplementary Note 4. Synthesis of Cu-anchored polymeric carbon nitride (Cu-PCN).

Cu-PCN samples were prepared by modifying our previously reported method[2]. In the standard process, urea (30 g) was dissolved in hot water (7 mL, 95 °C) under continuous magnetic stirring. An aqueous solution of $CuCl_2$ (1 mL, 0.1 M) was then added to the mixture to obtain a transparent solution. After being kept at 95 °C for 5 min under continuous stirring, the solution was immediately cooled down using an ice-water bath, leading to the formation of solid crystals. The resulting crystals was dried in an oven at 70 °C for 12 h, ground into powder and dried further at 100 °C for 2 h. Subsequently, it was calcined at 550 °C for 4 h (with a heating rate 5 °C/min) in a muffle furnace. The obtained pale-yellow Cu-PCN powder with a Cu loading of 0.53 wt.% (weight percentage) was collected for further use. The Cu loading in the sample can be easily adjusted by using $CuCl_2$ precursor solutions with different concentrations, including 0.025 M (0.13 wt.%), 0.05 M (0.26 wt.%) and 0.2 M (1.05 wt.%). Pure PCN samples were prepared by the direct polymerization of urea at 550 °C for 4 h (with a heating rate 5 °C/min) in a muffle furnace. The yield for all the polymerization processes was approximately 4 wt.%.

Supplementary Note 5. EXAFS fitting parameters.

The obtained XAFS data was processed in Athena (version 0.9.26) for background, pre-edge line and post-edge line calibrations. Then Fourier transformed fitting was carried out in Artemis (version 0.9.26). The k3 weighting, k-range of 3 - 12 $Å^{-1}$ and R range of 1 - 3 Å were used for the fitting of foil; k-range of 2 - 11 $Å^{-1}$ and R range of 1 - 3 Å were used for the fitting of sample. The four parameters, coordination number, bond length, Debye-Waller factor and E_0 shift (CN, R, σ^2, ΔE_0) were fitted without anyone was fixed, constrained, or correlated.

Supplementary Note 6. Moisture resistance performance and mechanism.

High humidity severely degrades the electrical output performance of conventional polymer-based TENGs. However, for the quaternized CNF based TENG, the electrical output increases with the increase of humidity, because the hydroxyl groups in the CNF can form hydrogen bonds with the water molecules in the high-humidity environment, so that the water molecules participate in the contact electrification together[3]. When the humidity is increased from 15% RH to 95%RH, the current of quaternized CNF based TENG increases from 2.9 μA to 18.6 μA (Supplementary Fig. 9a), showing an enhanced electrical output property under high humidity. In order to determine the mechanism during this process, IR spectra of quaternized CNF films after water absorption at different humidity were tested. The results show that the -OH absorption peak of quaternized CNF film that stay in 95% RH shift from 3339.1 cm^{-1} to 3348.1 cm^{-1} compared with the film that was in 15% RH (Supplementary Fig. 9b)[4].

Supplementary Note 7. Comparison of output of pure CNF-Cu-PCN@PVDF based and quaternized CNF-Cu-PCN@PVDF based TENGs.

The quaternized CNF-based TENG displays better electrical output performance than the pure CNF-based TENG because the quaternized CNF contains positive charges[5], as shown in Supplementary Fig. 10a and 10b. Pure CNF-PVDF fiber based TENG's output current and voltage are 4.1 μA and 84 V, respectively. The quaternized CNF-based TENG's output current and voltage were 10.4 μA and 227 V, respectively, showing that the presence of positive charges in the quaternary ammonium salt increases the TENG's electrical output. Additionally, for electronegative PVDF, films and electrospun fibers also significantly affect the electrical output of TENG. The electrical output of the quaternized CNF-PVDF film based TENG is 6.8 μA and 143 V, whereas that of the quaternized CNF-PVDF fiber based TENG is 10.4 μA and 227 V (Supplementary Fig. 10c and 10d).

Supplementary Note 8. Effect of high voltage generated by TENG on CO_2 dissociation.

The dissociation of CO_2 into carbon monoxide typically necessitates elevated temperatures and considerable energy input to surmount the activation energy barrier for the dissociation reaction. Generally, CO_2 dissociation is accomplished under conditions involving an electron beam or plasma discharge[6,7]. The TENG based on quaternized CNF-Cu-PCN@PVDF demonstrates the capability to generate a voltage of 405 V during operation, and notably, no electrostatic discharge was observed. To experimentally investigate the potential of the 405 V electrostatic voltage to dissociate CO_2, we arranged two fixed metal electrodes (no movement and contact during the reaction) within a sealed transparent chamber (as shown in Supplementary Fig. 12a and 12b). Direct current (DC) power was employed to apply a voltage of 410 V across the two iron plates, creating an electric field. As depicted in Supplementary Fig. 12c, no discernible peak corresponding to CO is observed in the GC spectrum after a 5-hour reaction, indicating that the CO products were generated from the contact electrocatalysis process of TENG rather than direct high-voltage dissociation.

Supplementary Note 9. Effect of tribolayer thickness on electrical output and CO_2RR of TENG.

To fabricate Cu-PCN@PVDF membranes with varying thicknesses, we initiated the process by preparing electrospun films. Initially, 0.88 g of PVDF powder was blended with 3.6 g of acetone and stirred at room temperature. Simultaneously, 0.88 mg Cu-PCN powder was combined with 2.4 g of DMF and sonicated for 10 min. Subsequently, the Cu-PCN/DMF mixture was gradually introduced into the PVDF/acetone blend, and stirring was sustained for 24 h. The resulting mixture (about 5 mL) was then subjected to the electrospinning process. To achieve diverse thicknesses, we varied the electrospinning times to 1 h, 2 h, 3 h, 4 h, and 5 h. It's important to highlight that, for the “4 cm×4 cm” tribolayer of Cu-PCN@PVDF, the catalyst mass within the membranes, corresponding to electrospinning times of 1 h, 2 h, 3 h, 4 h, and 5 h, are 0.17 mg, 0.34 mg, 0.51 mg, 0.68 mg, and 0.85 mg, respectively. The sample electrospun for 4 h, with a catalyst content of 0.68 mg, stands out as the superior specimen highlighted in the study. The thickness corresponding to the electrospinning time is shown in Supplementary Fig. 13a. Following that, we employed SEM to examine the cross-sections of Cu-PCN@PVDF membranes electrospun for varying durations, aiming to characterize their thickness, as illustrated in Supplementary Fig. 13b-f. The findings indicate a direct correlation between the spinning time and the thickness of the Cu-PCN@PVDF film, with an observable increase as the spinning time extends. Then, the impact of Cu-PCN@PVDF electrospun fiber thickness on TENG output current, CO production, and yield was investigated, as shown in Supplementary Fig. 13g. The outcomes reveal a positive correlation between the thickness of the Cu-PCN@PVDF tribolayer and the electrical output of the TENG, escalating from 56 μm to 297 μm. However, with further thickness increments, there is a subsequent decline in the electrical output, as shown by the blue line in Supplementary Fig. 13g. This observation is rationalized by the understanding that an excessively thick tribolayer impacts charge separation and recombination, consequently impeding the generation of induced charges[8,9].

The electrical output of the TENG plays a pivotal role in influencing the production of CO during electrocatalytic CO_2RR. Consequently, as the thickness of the tribolayer increases, there is an observed trend of initially rising and subsequently declining CO production, as shown by the green line in Supplementary Fig. 13g. Finally, we

calculated the CO yield, shown as the orange line in Supplementary Fig. 13g. The results show that with an increase in the thickness of the tribolayer, there is a notable decrease in CO yield. Since there is no triboelectric charge generated inside the PVDF fiber, the catalyst within does not contribute to CO_2RR. In this study, the precise content of fibers on the surface engaged in contact electrocatalysis for CO_2RR is difficult to determine accurately. Hence, we opted to calculate the CO yield using all catalysts present in the entire Cu-PCN@PVDF electrode. Consequently, as the catalyst mass increases, there is a corresponding decrease in CO yield. It's important to highlight that, while the catalytic yields of Cu-PCN@PVDF membranes with electrospinning times of 1 h, 2 h, and 3 h surpass those of 4 h, membranes that are excessively thin face a susceptibility to damage upon contact and separation from quaternized CNF. Considering the long-term application perspective, the fiber membrane with an electrospinning time of 4 h was judiciously chosen as the electronegative tribolayer for TENG preparation in this study.

Supplementary Note 10. Effect of tribolayer area on electrical output and CO_2RR of TENG.

We conducted a comparative analysis of the output current and CO production in TENGs with electrode geometric areas of "2 cm×2 cm", "3 cm×3 cm", "4 cm×4 cm", and "5 cm×5 cm", as illustrated in Supplementary Fig. 14a. The findings reveal a direct correlation between the geometric area of the electrode and both the current output and CO production of the TENG. The observed trend is logical, as a larger geometric area results in increased generation of frictional and induced charges within the same material, consequently leading to higher electrical output. This rise in charge signifies a greater number of electrons participating in the CO_2RR, thereby causing an escalation in CO production[10,11]. The entity photographs of Cu-PCN@PVDF electrodes with different geometric areas are shown in Supplementary Fig. 14b.

Supplementary Note 11. Device stability analysis.

To further investigate the stability of materials exposed to electrocatalytic CO_2RR, we analyzed the standard deviation (STDEV) of CO production and current changes during the cycling experiment, as shown in Supplementary Table 2. The outcomes reveal a standard deviation of 3.28 for CO production, signifying a minimal degree of dispersion. This suggests that the materials employed in catalyzing electrocatalytic CO_2RR exhibit high stability. Furthermore, prior to and following the reaction, the standard deviations of the TENG current are about 0.07 and 0.1, respectively. The low current fluctuations imply the stability of the TENG device.

Supplementary Note 12. Effect of high-humidity on CO_2 concentration.

During the contact-electro-catalytic CO_2RR experiments, the environmental humidity was regulated to 99% to ensure an ample supply of protons essential for the reduction reaction. At 99% RH, the CO_2 introduced into the sealed environment predominantly adsorbs onto the surface of the quaternized CNF membrane, assuming the form of carbonic acid. Furthermore, post-CO_2RR reaction, a significant portion of the excess CO_2 undergoes dissolution within droplets, taking the form of carbonic acid and adhering to the walls of the closed box (Supplementary Fig. 18a). The collected droplets were subjected to pH testing, as depicted in Supplementary Fig. 18b-d. The outcomes revealed discernible acidity in the water attached to the sealed box, unequivocally establishing the presence of carbonic acid.

Supplementary Note 13. Wind-driven TENG catalyzes CO_2 reduction in air.

In the present design, the catalytic system is achieved by integrating a fan and the wind-driven TENG device in a closed reactor, as shown in Supplementary Fig.22a and 22b. The fan was utilized to provide wind energy for TENG movement and was equipped with three speed modes (low, medium, and high). The wind speeds were measured using an anemometer (UT363) and determined to be 4 m/s, 5.8 m/s, and 8 m/s for the fan's low, medium, and high-speed modes, respectively (Supplementary Fig.22c-f). As a proof-of-concept, the wind direction was adjusted to be parallel to the surface of the TENG's tribolayer. During the operation, the quaternized CNF film moves up and down under the influence of the wind, making contact with the Cu-PCN@PVDF tribolayer. The real-time electrical output of the device was monitored by a computer and used to calculate the CO Faradaic efficiency (FE_{CO}).

To ensure the stability of the wind-driven TENG device, we reduced the distance between the upper and lower Cu-PCN@PVDF/Al electrodes to 3 cm, facilitating smoother contact between the two tribolayers, i.e. Cu-PCN@PVDF and quaternized CNF. Furthermore, we increased the thickness of the quaternized CNF film to 0.3 mm, bestowing the film with enhanced flexibility and potentially reducing the risk of breakage during operation. We then conducted a test on the real-time electrical output of the wind-driven TENG device over 14 h at a wind speed of 8 m/s. As shown in Supplementary Fig.22g, the electrical output of the TENG device remains relatively stable without significant attenuation.

To test the CO_2 reduction performance of the wind-driven TENG device, we carried out contact-electro-catalytic CO_2RR experiments under different wind speeds (4 m/s, 5.8 m/s, and 8 m/s). Prior to each test, the fan was activated and the reactor was purged with compressed air to remove any potential impurities. Then the reactor was sealed and the humidity inside was stabilized at 99% RH. The reaction lasted for 5 h after the TENG's electrical output was stabilized. The real-time electrical outputs of the wind-driven TENG are shown in Supplementary Fig.22h-j. The results indicate a gradual increase in the electrical output of the TENG with higher wind speeds, aligning with the greater vibration amplitude and frequency of the TENG tribolayer under these conditions. Afterward, CO production was detected by GC, and the FE_{CO} was calculated. The FE_{CO} values obtained at wind speeds of 4 m/s, 5.8 m/s, and 8 m/s were determined to be 91.86%, 92.10%, and 92.33%, respectively (Supplementary Fig.22k). Then, we

calculated the Faradaic efficiency of the by-product H_2 (FE_{H2}), as shown in Supplementary Fig.22l. It should be noted that the H_2 product cannot be accurately quantified due to its extremely low yields. For CO_2RR catalyzed by the wind-driven TENG device, the CO product attains a high FE of around 92%, which means that there are limited electrons available for H_2 production. In addition, compared to the CO detection using a methanizer and a GC flame ionization detector, the H_2 detection by a thermal conductivity detector has lower sensitivity. Therefore, the obtained FE_{H2} can only be used as a reference.

Supplementary Figures

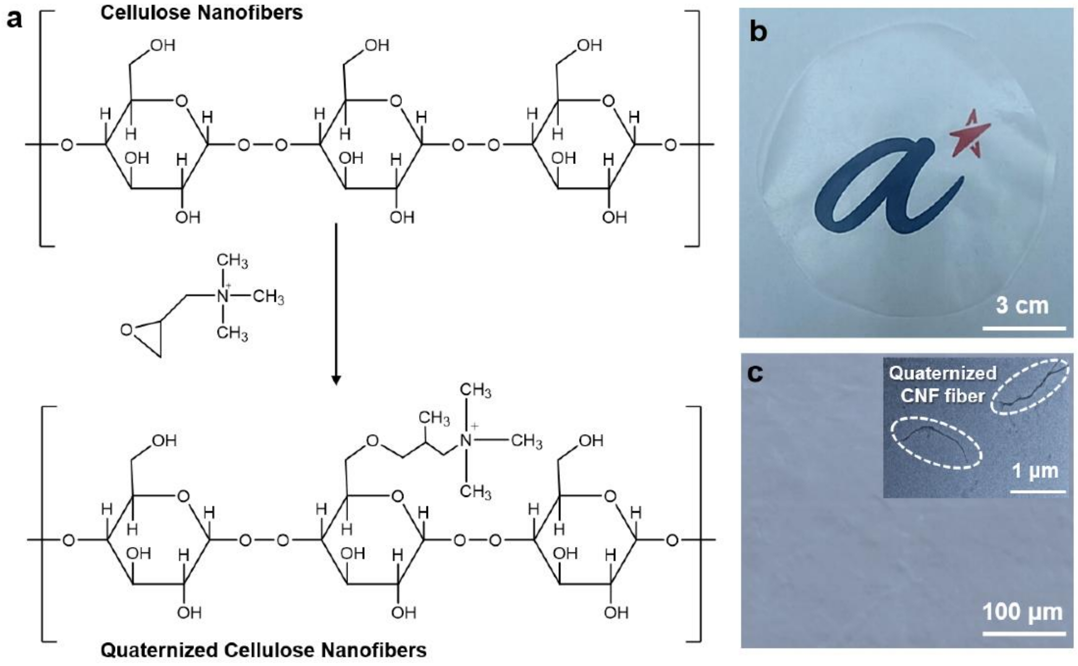

Supplementary Fig. 1 a Schematic diagram of the preparation process of quaternized CNF. **b** Physical photo of quaternized CNF film. **c** SEM and TEM of quaternized CNF.

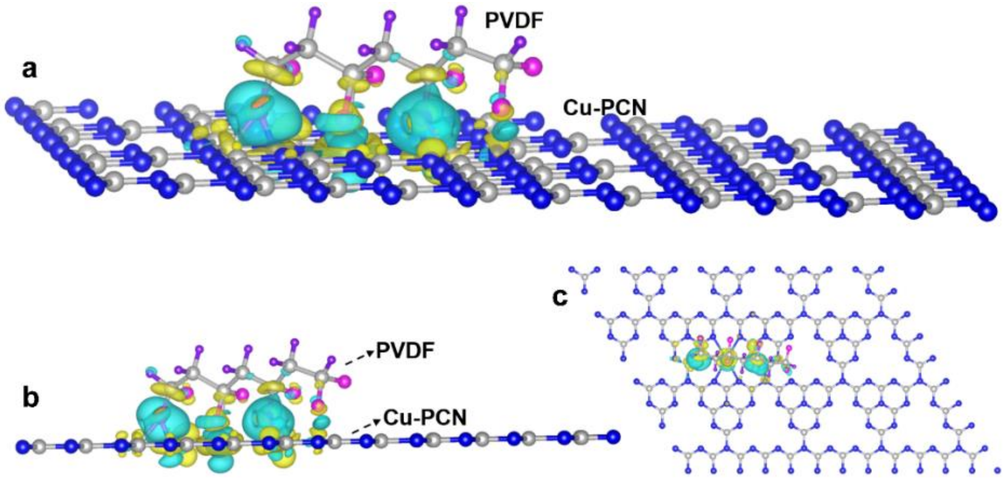

Supplementary Fig. 2 Simulated charge distribution near the PVDF and Cu-PCN surfaces during contact electrification. a Main view, **b** side view and **c** top view.

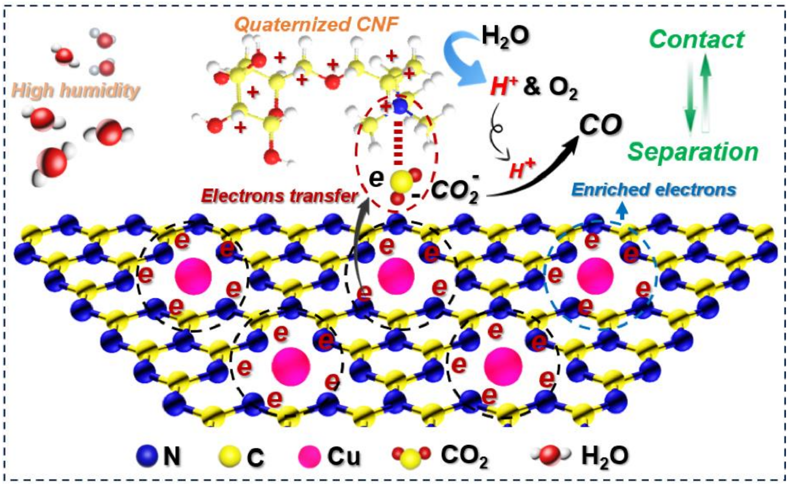

Supplementary Fig. 3 Schematic diagram of electron transfer on the Cu-PCN surface of the catalyst during the contact electrocatalytic CO_2RR process.

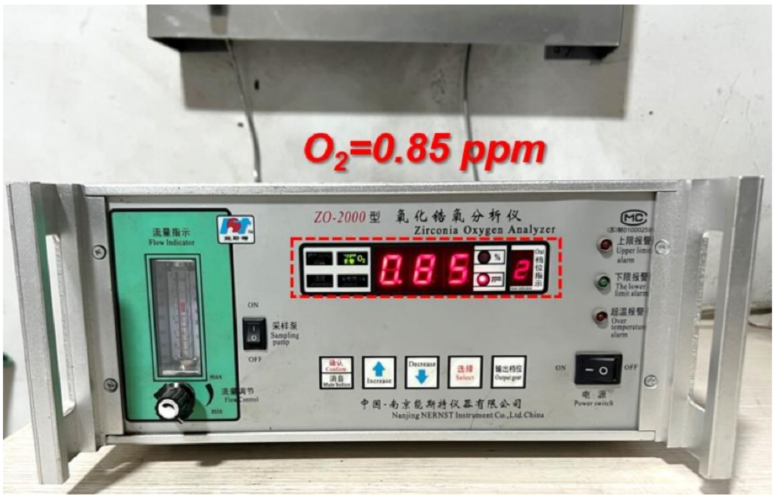

Supplementary Fig. 4 Photo of oxygen concentration after CO_2RR tested by zirconia oxygen analyzer.

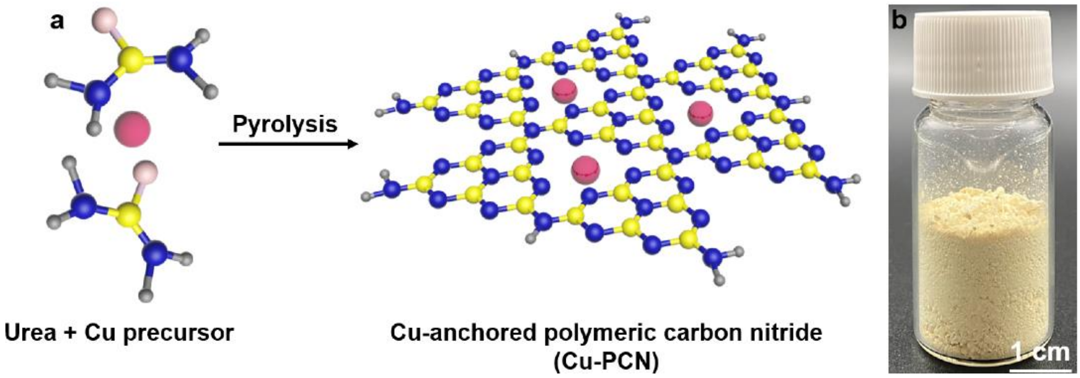

Supplementary Fig. 5 **a** Schematic diagram of the preparation process of Cu-PCN. **b** Physical photo of Cu-PCN powder.

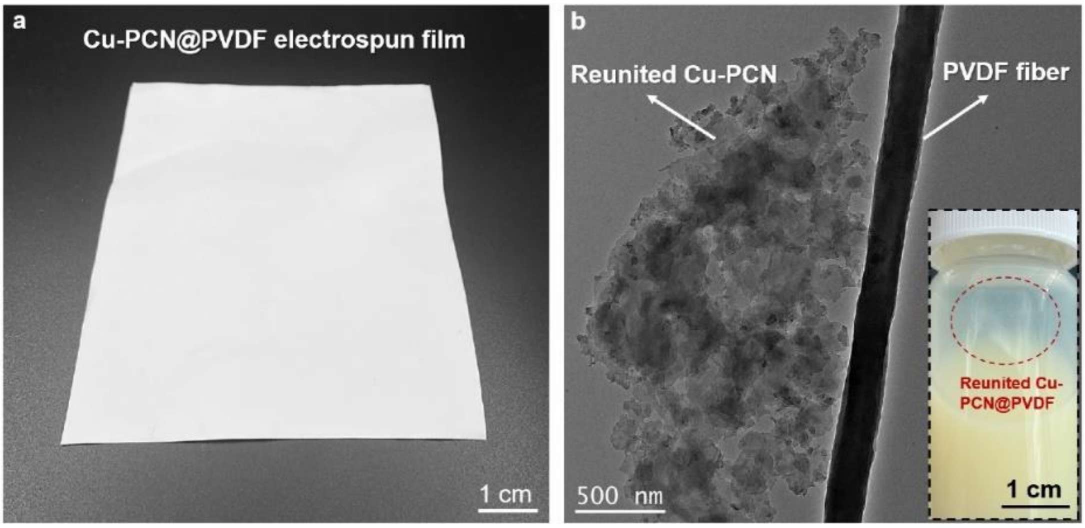

Supplementary Fig. 6 a Physical photo of Cu-PCN@PVDF electrospun membrane. **b** TEM image of reunited Cu-PCN and reunited Cu-PCN@PVDF electrospinning solution.

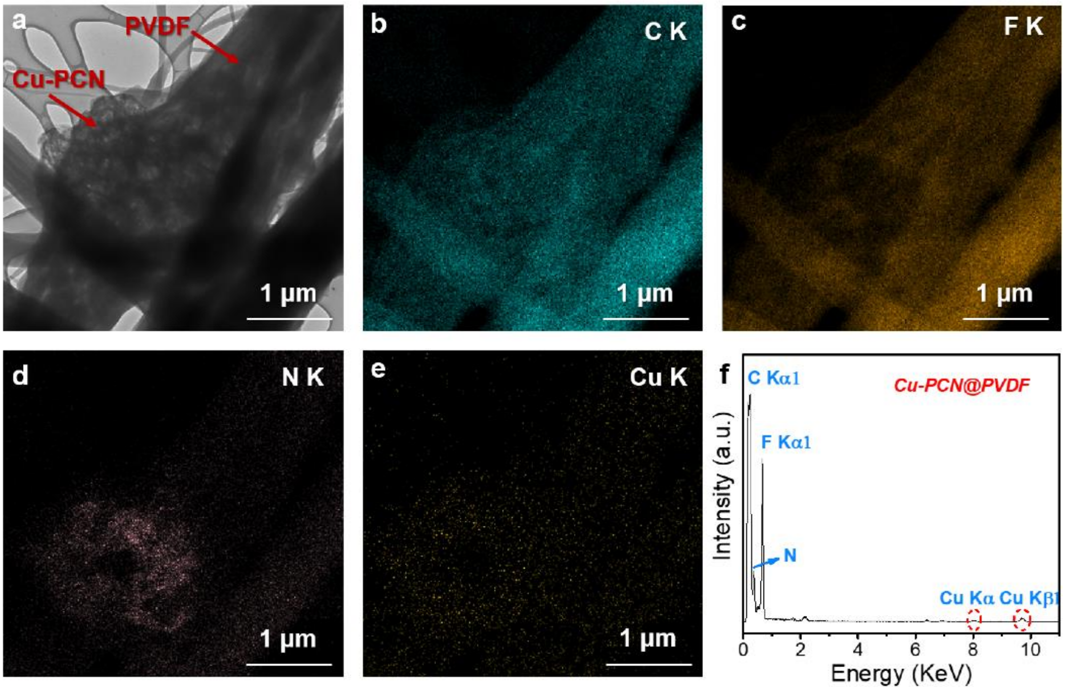

Supplementary Fig. 7 a TEM photo of Cu-PCN@PVDF. **b** Carbon element, **c** fluorine element, **d** nitrogen element and **e** copper element. **f** EDS of Cu-PCN@PVDF.

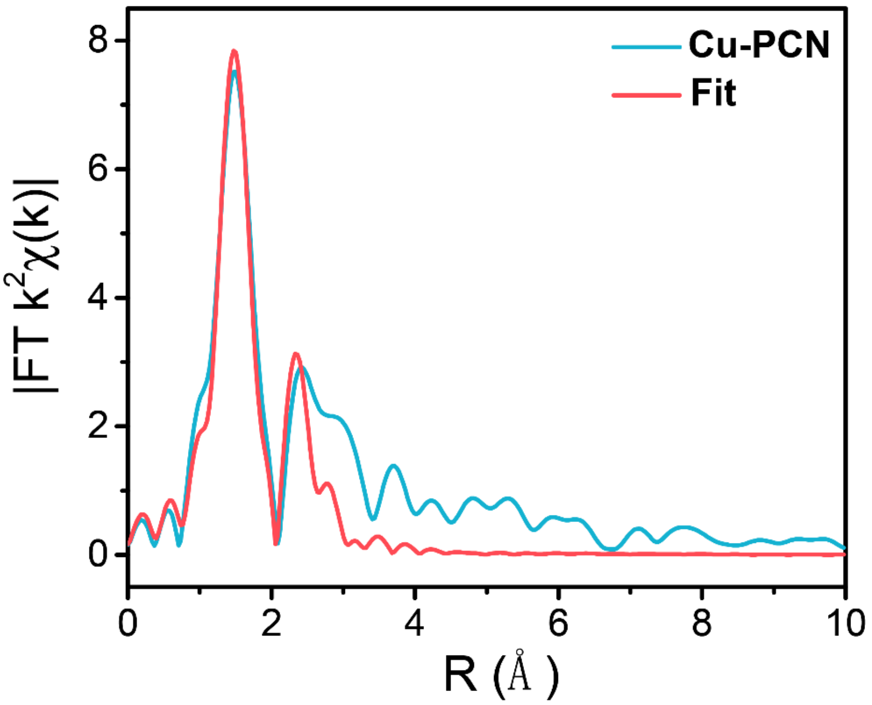

Supplementary Fig. 8 Fourier-transformed magnitudes of the k3-weighted Cu K-edge EXAFS spectra from Cu-PCN and the corresponding fitting analysis.

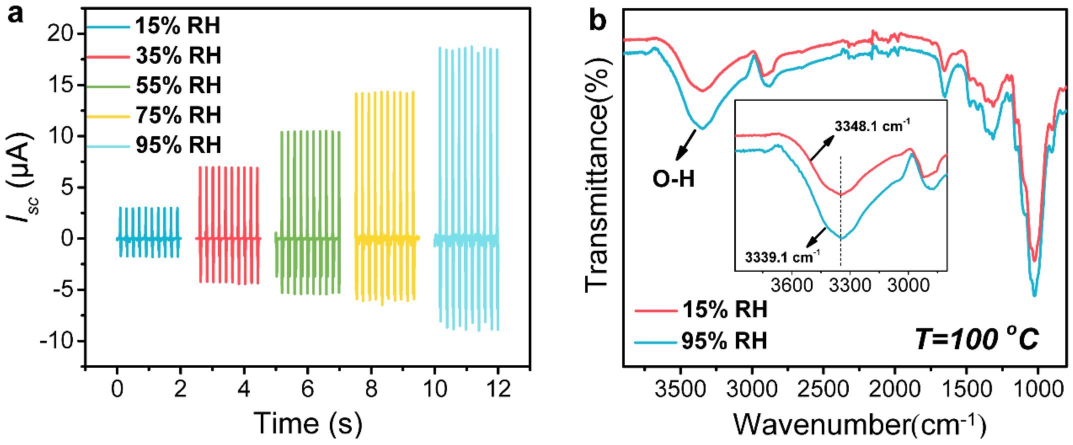

Supplementary Fig. 9 a Current output of quaternized CNF-Cu-PCN@PVDF-based TENG under different humidity. **b** 100°C-IR spectra of quaternized CNF film at 15%RH and 95%RH.

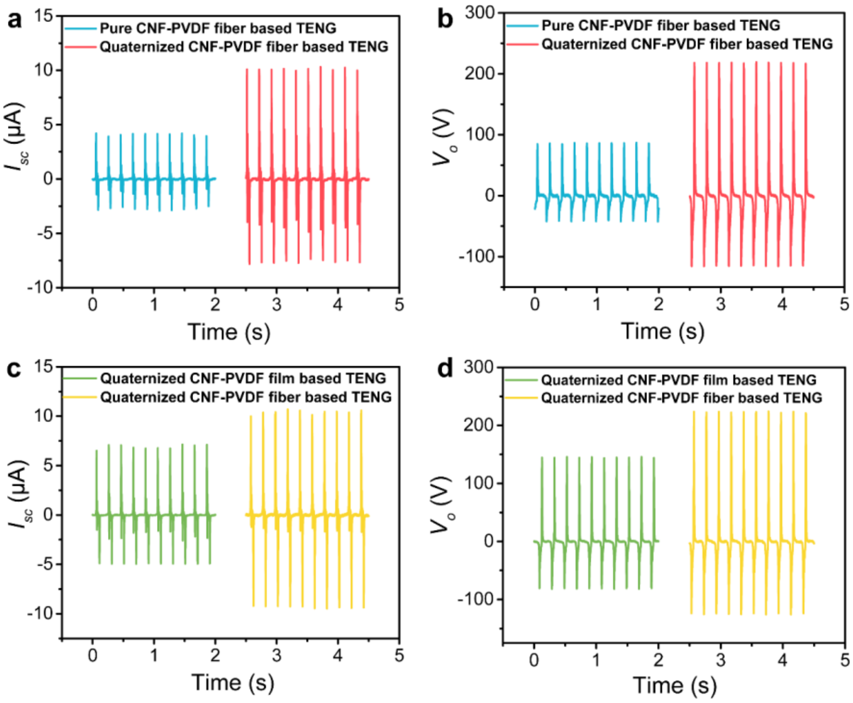

Supplementary Fig. 10 **a** Current and **b** voltage comparison of pure CNF-Cu-PCN@PVDF based and quaternized CNF-Cu-PCN@PVDF based TENGs. **c** Current and **d** voltage comparison of quaternized CNF-Cu-PCN@PVDF fiber-based and quaternized CNF-Cu-PCN@PVDF film-based TENGs.

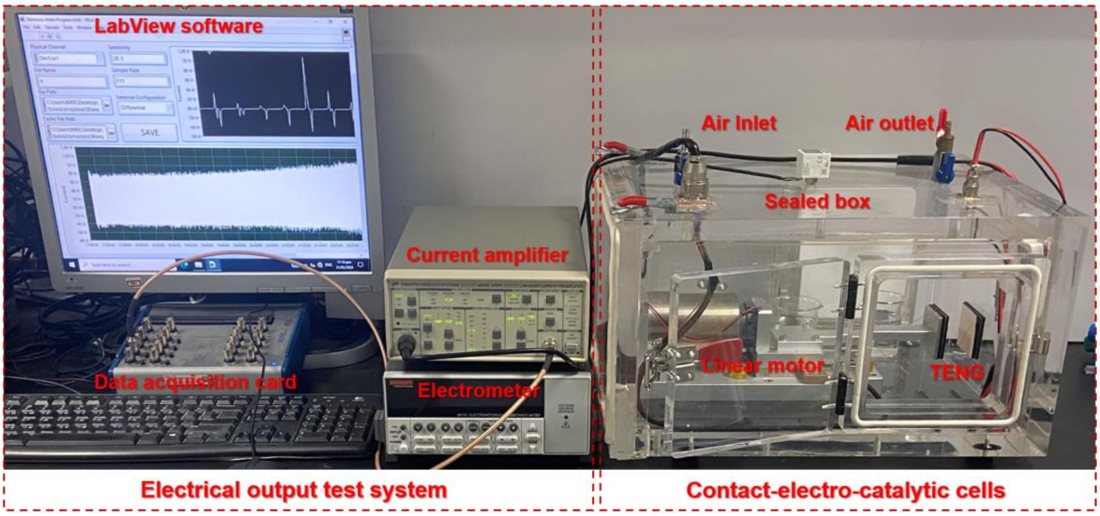

Supplementary Fig. 11. Photo of the contact-electro-catalytic CO_2RR test system.

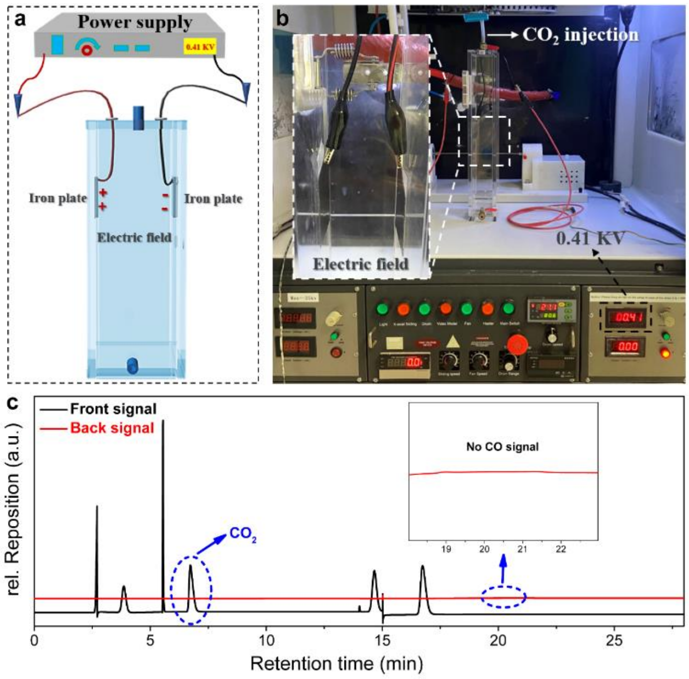

Supplementary Fig. 12 a Schematic and **b** photo of the device for CO_2 dissociation at 410 V. **c** GC spectrum of CO_2 dissociation.

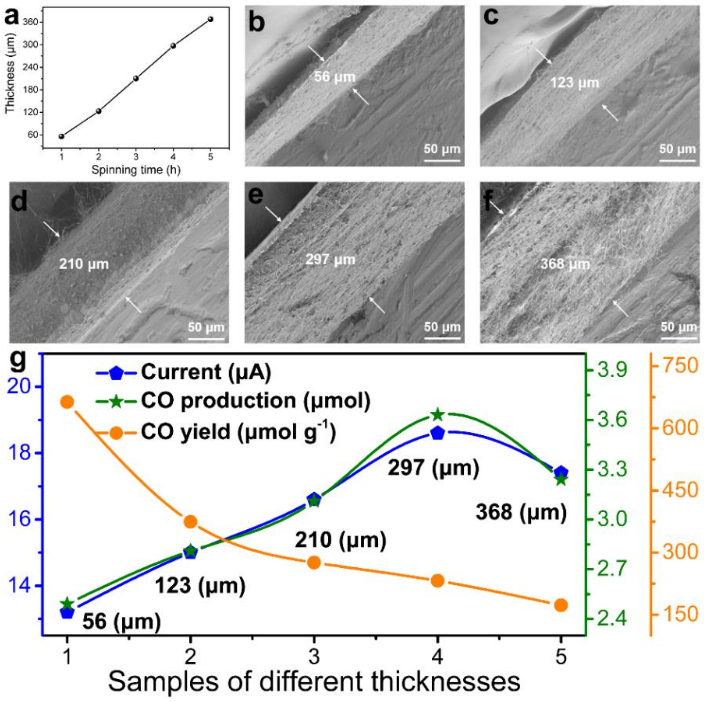

Supplementary Fig. 13 a Effect of electrospinning time on fiber membrane thickness. SEM cross-sectional photos of Cu-PCN@PVDF films electrospun for **b** 1 h, **c** 2 h, **d** 3 h, **e** 4 h and **f** 5 h. **g** Investigating the impact of Cu-PCN@PVDF electrospun fiber thickness on TENG output current (blue line), CO production (green line), and yield (orange line).

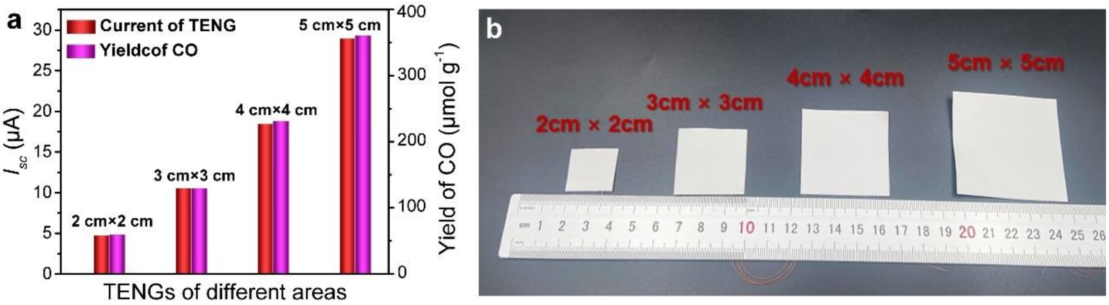

Supplementary Fig. 14 a Comparison of current output and CO yield of TENGs with different geometric areas. **b** Entity photographs of Cu-PCN@PVDF electrodes with different geometric areas.

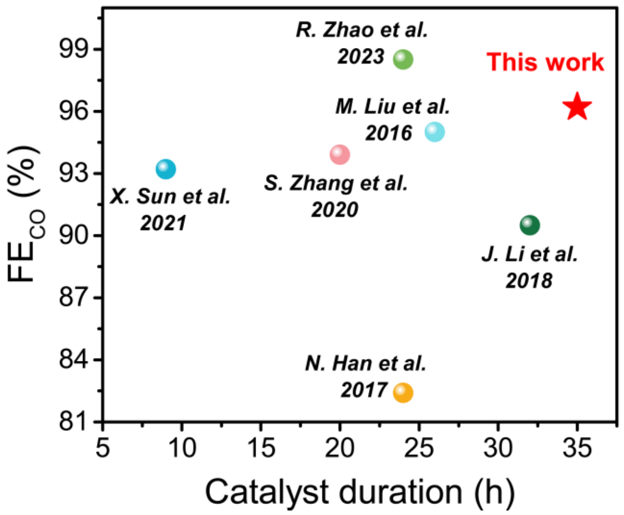

Supplementary Fig. 15 CO Faradaic efficiency (FE_{CO}) and catalyst duration of contact-electro-catalytic CO_2RR versus that of traditional electrocatalytic techniques.

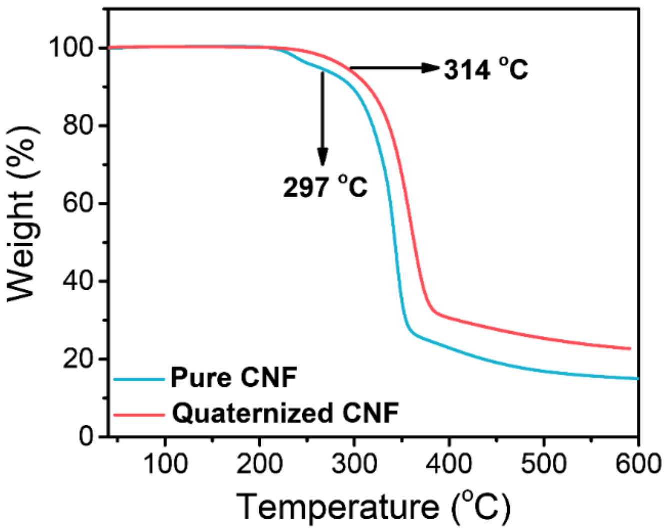

Supplementary Fig. 16 TGA comparison of pure CNF and quaternized CNF films.

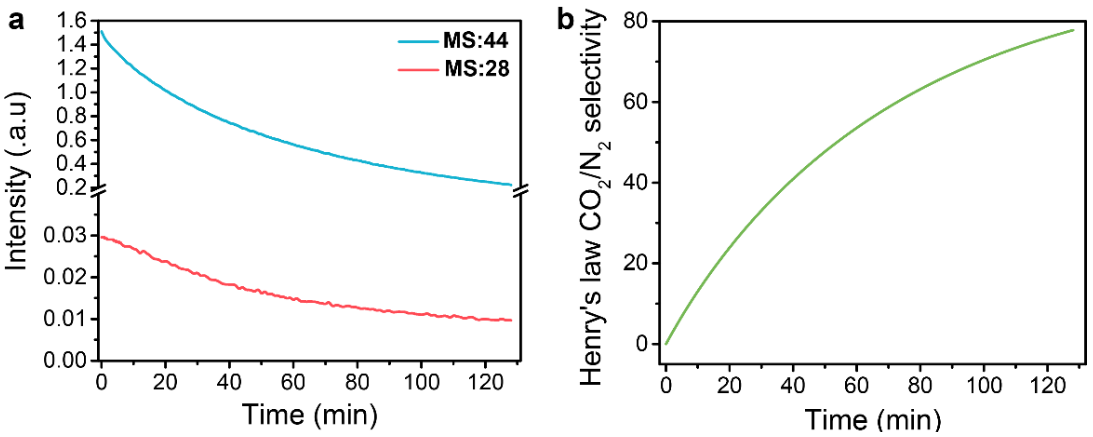

Supplementary Fig. 17 a Spectra of CO_2 (MS:44) and N_2 (MS:28) collected by MS within 130 min**. b** Henry's law CO_2/N_2 selectivity.

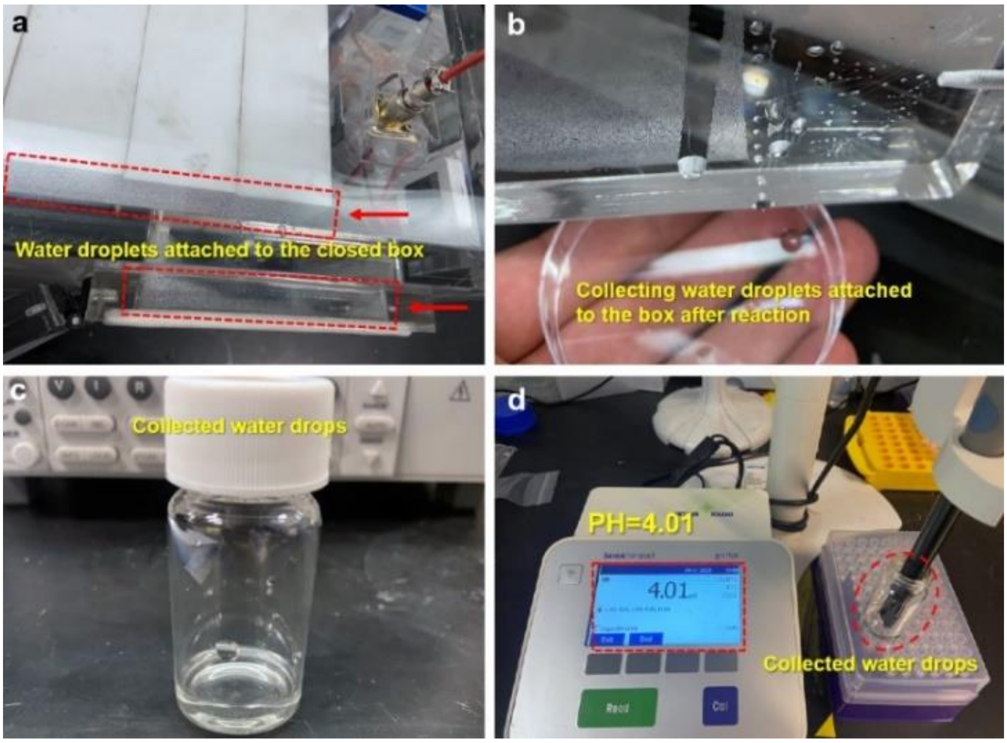

Supplementary Fig. 18 a Photo of water droplets attached to the wall of a closed box. **b, c** Photo of collected water droplets attached to the wall of the closed box. **d** Photo of collected water droplets being tested for pH.

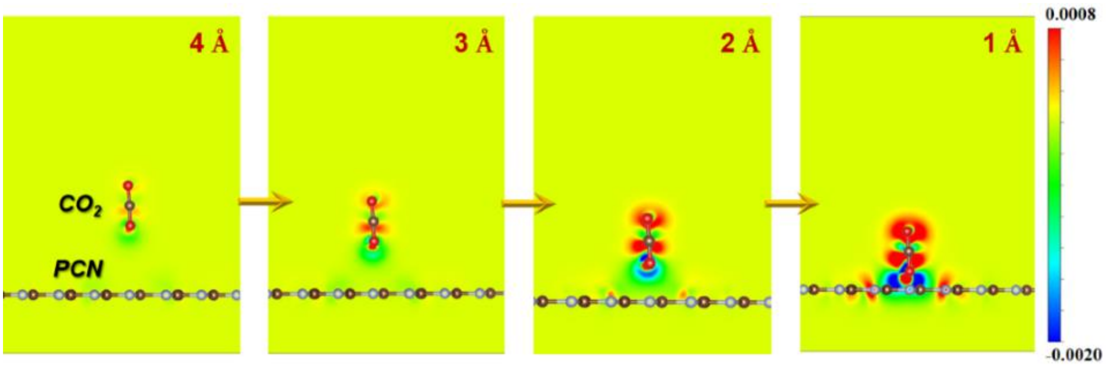

Supplementary Fig. 19 Charge distribution near PCN surface during contact.

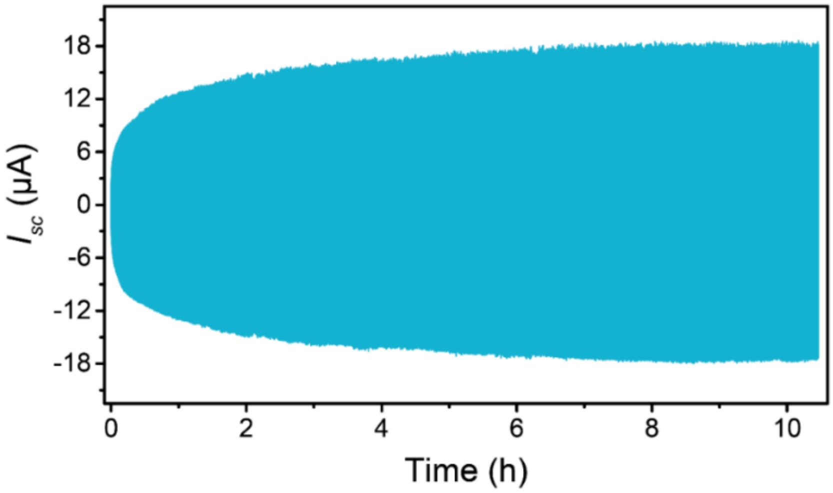

Supplementary Fig. 20 Real-time current output of quaternized CNF-Cu-PCN@PVDF based TENG under Ar environment.

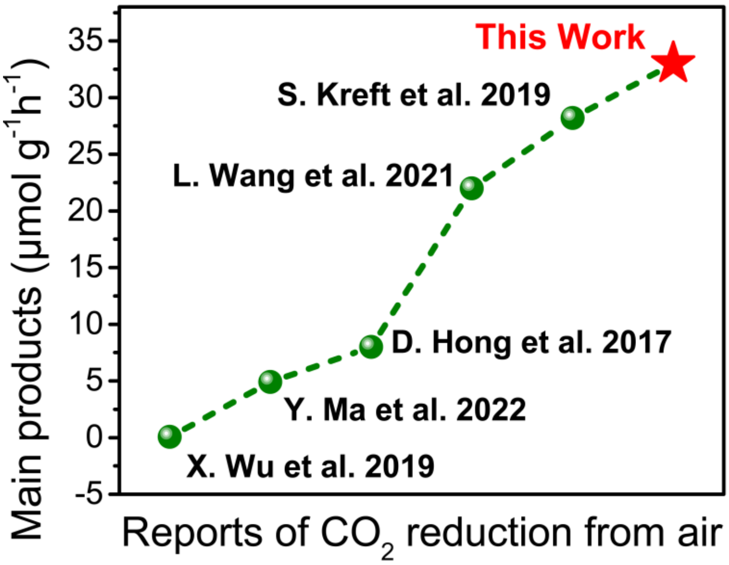

Supplementary Fig. 21 CO yield of contact-electro-catalytic CO_2RR in air compared with conventional photocatalysis.

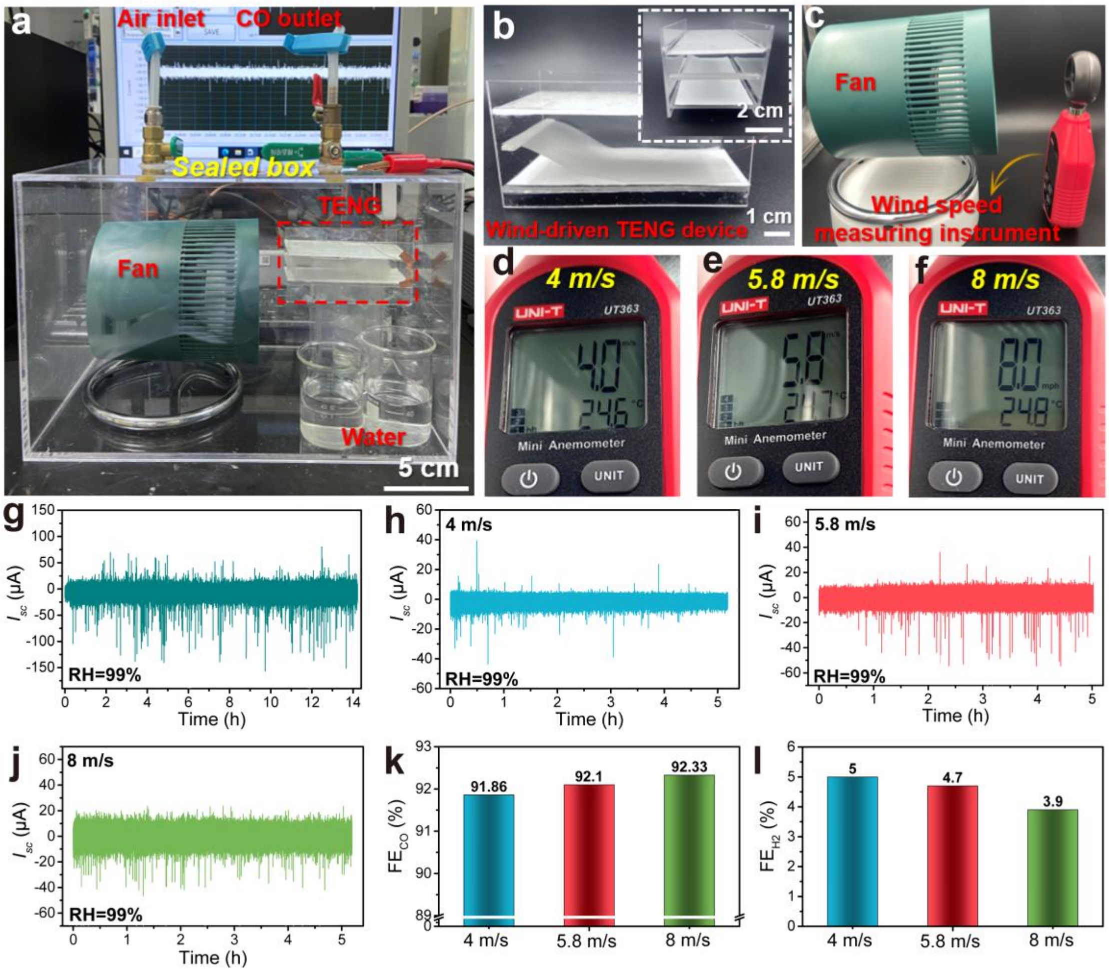

Supplementary Fig. 22 a Photograph of wind-driven TENG device for contact-electro-catalytic CO_2 reduction in air. **b** Front view and side view (inset) photos of the wind-driven TENG device. **c** Photograph depicting the wind speed measuring instrument gauging the fan's airflow at different speeds. Images captured by the speed measuring instrument showing the fan's airflow at **d** low (4 m/s), **e** medium (5.8 m/s), and **f** high (8 m/s) speeds. **g** Electrical output stability test of wind-driven TENG under wind speed of 5.8m/s. The electrical output of the wind-driven TENG under airflow velocities of **h** 4m/s, **i** 5.8m/s and **j** 8m/s during contact-electro-catalytic CO_2RR in air. **k**, **l** Comparison of FE_{CO} and FE_{H2} obtained under these three airflow speeds. FE is the calculated result of a single test. The FE_{H2} can only be used as a reference because the yields of H_2 products are extremely low and thus cannot be accurately determined by GC.

Supplementary Tables

Supplementary Table 1 EXAFS fitting parameters at the Cu K-edge (S_0^2=0.88)

Sample	Cu foil	Cu
Path	Cu-Cu	Cu-N
C.N.	12*	5.4±1.8
R (Å)	2.54±0.01	1.95±0.02
$\sigma^2 \times 10^3$ (Å^2)	8.1±2.9	4.7±3.0
ΔE (eV)	4.0±0.6	2.6±4.1
R factor	0.002	0.018

C.N.: coordination numbers; R: bond distance; σ^2: Debye-Waller factors; ΔE: the inner potential correction. R factor: goodness of fit. * Fitting with fixed parameter.

Supplementary Table 2 Analyzing CO yield and TENG current across 7 cycles using analysis of variance.

Cycles	1	2	3	4	5	6	7	Standard deviation
Yield (μmolg^{-1})	239	231	239	234	236	241	234	3.282607227
I_{sc1} (μA)	18.6	18.5	18.6	18.5	18.6	18.7	18.5	0.069985421
I_{sc2} (μA)	16.5	16.4	16.6	16.3	16.5	16.4	16.6	0.103015751

Supplementary Table 3 Quaternized CNF-Cu-PCN@PVDF based TENG catalyzes CO_2RR in CO_2/Ar mixed gas with 20% CO_2 content.

Sample	Standard	TENG
Injection volume/mL	0.25	1
Container volume/L		5
Catalyst weight/mg		0.68
Q/mC		31.5
Time/h		5
Production rate/nmolh^{-1}		31.42
EF_{co}		96.24%

Peak area	426.26	6
Yield/nmol	2.23	157.1

Supplementary Table 4 Comparison of FE_{CO} and FE_{CO} between contact-electro-catalytic CO_2RR and state-of-the-art electrocatalytic CO_2RR using analysis of variance.

	N. Han et al.[12]	J. Li et al.[13]	M. Liu et al.[14]	X. Sun et al.[15]	S. Zhang et al.[16]	R. Zhao et al.[17]	This Work
Cycle-1	82.9	90.5	94.8	94.8	94.1	99.5	96.2
Cycle-2	81.9	90	93.5	93.5	93.1	99.1	96.5
Cycle-3	83.1	90.3	95.1	93.6	92.5	99	97
Cycle-4	80.5	89	95.9	92.5	94.5	99.1	97.2
Cycle-5	83.6	90.1	95.5	92.1	94.6	98	95.1
Cycle-6	82.4	92	95.1	93.2	91.1	98.2	95.5
Cycle-7	82.5	91.7	95.3	92.8	97.2	97.5	96.2
STDEV	0.93	0.96	0.7	0.82	1.78	0.69	0.69
Average	82.4	90.5	95	93.2	93.9	98.5	96.2

Supplementary Table 5 Quaternized CNF-Cu-PCN@PVDF based TENG catalyzes CO_2RR in air.

Sample	Standard	TENG
Injection volume/mL	0.25	1
Container volume/L		5
Catalyst weight/mg		0.68
Q/mC		48.4
Time/h		10.5
Production rate/nmolh^{-1}		22.44
EF_{co}		93.95%
Peak area	426.26	9
Yield/nmol	2.23	235.6

Supplementary References

1. Quinlan, P. J., Tanvir, A. & Tam, K. C. Application of the central composite design to study the flocculation of an anionic azo dye using quaternized cellulose nanofibrils. *Carbohydr. Polym.* **133**, 80-89, (2015).
2. Jiang, W. *et al.* Integration of Single-Atom Catalyst with Z-Scheme Heterojunction for Cascade Charge Transfer Enabling Highly Efficient Piezo-Photocatalysis. *Adv. Sci.* 2303448 (2023).
3. Sun, W. *et al.* Humidity-resistant triboelectric nanogenerator and its applications in wind energy harvesting and self-powered cathodic protection. *Electrochim. Acta.* **391**, 138994 (2021).
4. Wang, N. *et al.* New hydrogen bonding enhanced polyvinyl alcohol based self-charged medical mask with superior charge retention and moisture resistance performances. *Adv. Funct. Mater.* **31**, 2009172 (2021).
5. Rana, S. S. *et al.* Cation functionalized nylon composite nanofibrous mat as a highly positive friction layer for robust, high output triboelectric nanogenerators and self-powered sensors. *Nano Energy* **88**, 106300 (2021).
6. Navascués, P., Cotrino, J., González-Elipe, A. R. & Gómez-Ramírez, A. Plasma assisted CO_2 dissociation in pure and gas mixture streams with a ferroelectric packed-bed reactor in ambient conditions. *Chem. Eng. J.* **430**, 133066 (2022).
7. Li, S. *et al.* Triboelectric Plasma CO_2 Reduction Reaching a Mechanical Energy Conversion Efficiency of 2.3. *Adv. Sci.* **9**, 202201633 (2022).
8. Kim, D. W., Lee, J. H., You, I., Kim, J. K. & Jeong, U. Adding a stretchable deep-trap interlayer for high-performance stretchable triboelectric nanogenerators. *Nano Energy* **50**, 192-200 (2018).
9. Kim, M. P. *et al.* Interfacial polarization-induced high-k polymer dielectric film for high-performance triboelectric devices. *Nano Energy* **82**, 105697 (2021).
10. Yang, W. *et al.* Fundamental research on the effective contact area of micro-/nano-textured surface in triboelectric nanogenerator. *Nano Energy* **57**, 41-47 (2019).
11. Zhang, R. & Olin, H. Material choices for triboelectric nanogenerators: A critical

review. *EcoMat* **2**, e12062 (2020).

12. Han, N. *et al.* Supported cobalt polyphthalocyanine for high-performance electrocatalytic CO_2 reduction. *Chem* **3**, 652-664, (2017).
13. Li, J. *et al.* Efficient electrocatalytic CO_2 reduction on a three-phase interface. *Nat. Catal.* **1**, 592-600 (2018).
14. Liu, M. *et al.* Post-synthetic modification of covalent organic frameworks for CO_2 electroreduction. *Nat. Commun.* **14**, 3800 (2023).
15. Sun, X.-C. *et al.* Au^{3+} species-induced interfacial activation enhances metal–support interactions for boosting electrocatalytic CO_2 reduction to CO. *ACS Catal.* **12**, 923-934 (2021).
16. Zhuang, S. *et al.* Hard-sphere random close-packed Au (47) Cd (2) (TBBT) (31) nanoclusters with a faradaic efficiency of up to 96 % for electrocatalytic CO_2 reduction to CO. *Angew. Chem., Int. Ed.* **59**, 3073-3077 (2020).
17. Zhao, R. *et al.* Partially nitrided Ni nanoclusters achieve energy-efficient electrocatalytic CO_2 reduction to CO at ultralow overpotential. *Adv. Mater.* **35**, e2205262 (2023).
